# Single‐cell sequencing reveals the heterogeneity and intratumoral crosstalk in human endometrial cancer

**DOI:** 10.1111/cpr.13249

**Published:** 2022-05-13

**Authors:** Zhicheng Yu, Jun Zhang, Qi Zhang, Sitian Wei, Rui Shi, Rong Zhao, Lanfen An, Richard Grose, Dilu Feng, Hongbo Wang

**Affiliations:** ^1^ Department of Obstetrics and Gynecology, Union Hospital, Tongji Medical College Huazhong University of Science and Technology Wuhan People's Republic of China; ^2^ Centre for Tumour Biology, Barts Cancer Institute Queen Mary University of London London UK; ^3^ Clinical Research Center of Cancer Immunotherapy Wuhan People's Republic of China

## Abstract

**Background:**

Endometrial cancer (EC) is one of the most common gynecologic malignancies with increasing morbidity. Cell–cell and cell‐matrix interactions within the tumour microenvironment (TME) exert a powerful influence over the progression of EC. Therefore, a comprehensive exploration of heterogeneity and intratumoral crosstalk is essential to elucidate the mechanisms driving EC progression and develop novel therapeutic approaches.

**Methods:**

4 EC and 2 normal endometrium samples were applied for single‐cell RNA sequencing (scRNA‐seq) analysis. In addition, we also included the public database to explore the clinical benefits of the single cell analysis.

**Results:**

9 types of cells were identified with specific expression of maker genes. Both the malignant epithelial cells and cells comprising the immune microenvironment displayed a high degree of intertumoral heterogeneity. Notably, the proliferation T cells also showed an exhausted feature. Moreover, the malignant cells may induce an immunosuppressive microenvironment through TNF‐ICOS pair. Cancer‐associated fibroblasts (CAFs) were divided into four subsets with distinct characteristics and they maintained frequent communications with malignant cells which facilitating the progression of EC. We also found that the existence of vascular CAF (vCAF) may indicate a worse prognosis for EC patients through integrating TCGA database.

**Conclusion:**

The TME of human EC remains highly heterogeneous. Out finding that malignant cells interact closely with immune cells and vCAFs identifies potential therapeutic targets.

## Graphical abstract

In order to comprehensively explore the heterogeneity and intra‐tumoral crosstalk of human endometrial cancer, we profiled 41,358 single cells of human endometrial cancer and normal endometrial tissues. The results showed that the tumour microenvironment of human ECs remains highly heterogeneity. Malignant cells interact closely with immune cells and vCAFs which may indicate potential therapeutic targets.

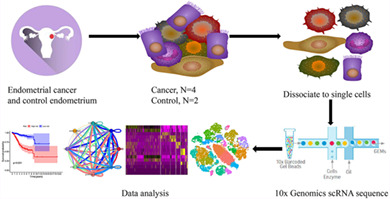

## 1 INTRODUCTION

Endometrial cancer (EC) is one of the most common malignant gynaecological cancers with increasing morbidity. Although most patients have a relatively favourable 5‐years survival as high as 80%, patients progress to the terminal stage quickly upon relapse.^1^, ^2^ The prognosis of EC has remained constant for years due to the fixed treatment mode, although numerous studies have been designed to reveal the biological characteristics and pathogenesis.

Tumours are complex ecosystems composed of different cell types, such as epithelial cells, immune cells, and stromal cells.^3^ The molecular subtypes identified based on the bulk sequencing of EC have been divided into four specific expression patterns that are closely related to disease prognosis.^4^ This specific expression pattern was partially attributed to the distinct proportions of various cell types in the tumour microenvironment (TME), indicating that the heterogeneity of EC is vital for disease progression. In addition, a prominent desmoplastic stroma in the TME, which is mainly composed of cancer‐associated fibroblasts (CAFs), is a hallmark of a worse prognosis for patients with EC.^5^ Therefore, a deeper understanding of the heterogeneity and intratumor crosstalk of distinct cells in the TME would help to identify more efficacious therapeutic targets for EC.

Single‐cell RNA sequencing (scRNA‐seq) has emerged as a powerful tool to reveal heterogeneity and cellular communication in a number of cancers, including breast cancer, lung cancer, head and neck cancer, pancreatic ductal adenocarcinoma and liver cancer.^6^, ^7^, ^8^, ^9^, ^10^ Salient anti‐tumour effects have been achieved when blocking the interaction between different cells identified by scRNA‐seq.^11^ However, the heterogeneity and intratumoral crosstalk of EC remain poorly elucidated at single‐cell resolution.

In this study, we profiled the transcriptome of 41,358 single cells from four EC tissues and two normal endometrial clinical samples based on 10× Genomics scRNA‐seq to elucidate the heterogeneity and intratumoral crosstalk in EC.

## 2 MATERIALS AND METHODS

### 2.1 EC and control samples

Four EC tissues and two control tissue samples were collected from Union Hospital, Tongji Medical College, Huazhong University of Science and Technology approved by the Institutional Review Board (2020‐S218) and patients enrolled in the study provided written informed consent. No patients received treatment prior to surgery, such as chemotherapy or radiotherapy. The control normal endometrium was obtained from patients who underwent hysterectomy due to nonmalignant gynaecological diseases. In addition, two patients with EC were excluded: patient EC1 was concurrently diagnosed with high‐grade serous ovarian carcinoma, and patient EC5 was not included because of an unsatisfactory number of cells captured.

### 2.2 Preparation of single‐cell suspensions and quality control

The freshly resected tissues were divided into two equal parts: one was prepared for subsequent single‐cell sequencing, and the other was processed for pathological diagnosis and immunohistochemical studies. We first washed the tissue with 1× DPBS (calcium‐ and magnesium‐free) twice and cut the tissue into approximately 2 mm pieces with ophthalmic scissors to prepare the single‐cell suspension. Second, 3 ml of collagenase I (1 mg/ml) was added to sufficiently digest the tissues for 50 min. Then, the cell suspension was filtered through a 70 μm cell strainer and centrifuged for 7 min at 300 *g*. Finally, the sediment was washed twice with precooled 1× DPBS containing 0.04% BSA after removing the supernatant. Dead cells were eliminated by excluding Sytox‐positive (Dead Cell Removal Kit, Miltenyi Bio.tec) cells according to the manufacturer's instructions, which increased the efficiency of sorting live cells for subsequent library construction and sequencing. The quality control of the cell suspension was estimated using a Countess II Automated Cell Counter. Eligible samples were defined as containing greater than 85% of living cells and a density greater than 1 × 10^6^ cells/ml.

### 2.3 Gene expression library construction and sequencing

The gene expression library was constructed according to the instructions of the 10× Genomics Chromium single‐cell kit. The libraries were then pooled and

sequenced using the Illumina NovaSeq 6000 platform.

### 2.4 Generation and preprocessing of single‐cell transcriptomes

The primary row data were converted to fastq format using the Illumina bcl2fastq converter and filtered to obtain clean data. The criteria included the following: 1, removal of polyA reads, 2, removal of reads containing more than 3 indeterminate bases, and 3, removal of low‐quality reads (the number of bases with a Q value less than or equal to 5 that accounted for more than 20% of the total reads). Then, the clean data were processed using Cell Ranger software (version 4.0.0) provided by 10× Genomics to demultiplex cellular barcodes and align valid barcodes, and STAR was used to align the reads with the reference genome (GRCh38‐2020‐A). The gene expression pattern was measured by determining unique molecular identifier (UMI) counts using Cell Ranger (Figure S2A,B).

Then, the multiplets and low‐quality cells were identified and filtered. Scrublet software was applied to predict multicellular barcodes and remove multicellular barcodes (Figure S2D). Low‐quality cells were excluded when 20% or more UMIs were mapped to mitochondrial genes to avoid the effect of apoptotic or lytic cells (Figure S2E). Next, we used Seurat to remove foreign cells. A gene with expression in more than 3 cells was considered expressed, and each cell was required to have at least 200 expressed genes. After strict quality control was performed, 41,358 single cells were detected in the downstream analysis in this study. Then, the gene expression data were normalized using the Seurat package with the normalization method “LogNormalize” to reduce the discrete number of gene expression counts. Finally, the correct transcriptome expression matrix was generated for subsequent analysis.

### 2.5 Dimensionality reduction, clustering and annotation

Highly variable genes (HVGs) were generated using the Seurat “Find Variable Features” function with default parameters except for selection.method=“vst” (Figure S2F). For clustering, HVGs were selected, subjected to principal component analysis (PCA) and the top 30 significant principal components (PCs) were selected to perform uniform manifold approximation and projection (UMAP) dimensionality reduction (Figure S2C). Cells were clustered with the Find Clusters function (dims.use = 1:30, resolution = 0.5) and were visualized in two dimensions using UMAP. Then, the SingleR R package with reference to Blueprint and the Human Primary Cell Atlas transcriptomic datasets were applied to annotate each cell cluster.^12^, ^13^ The Seurat alignment method canonical correlation analysis (CCA) was applied for the integrated analysis of datasets.

### 2.6 Identifying malignant cells with an InferCNV analysis based on scRNA sequencing data

The copy number variation in the four patients with EC was calculated from single‐cell transcriptomic profiles using InferCNV.^14^ Epithelial cells from normal endometrial tissue were selected as references. Briefly, CNV scores were computed in a sliding window equal to 101 for each chromosome with default parameters. Then, the expression matrix of reference and observation samples were combined for subsequent unsupervised *K*‐means clustering to identify the malignant subcluster. The variance was calculated based on each score derived from InferCNV to normalize the background noise. Finally, the subclusters with relatively higher CNV scores were considered malignant cells. A total of 2334 epithelial cells were identified as nonmalignant cells, and 16,050 epithelial cells were considered malignant cells.

### 2.7 Estimating the cycling cells

We first calculated the G1/M and G2/M scores for each cell by analysing a relevant gene set to estimate the cycling cells.^15^ Second, the cutoff value to distinguish high cycling cells from low cycling cells was considered the median plus 2 MAD (median absolute deviation).^6^ Briefly, cells were deemed to be high cycling cells if they had higher G1/M or G2/S scores, and low cycling cells were those with lower G1/M or G2/S scores. Finally, 6695 cells were determined to be high cycling cells, and 34,560 cells were determined to be low cycling cells. For epithelial cells, 4050 cells were regarded as high cycling cells, and 14,334 cells were regarded as low cycling cells.

### 2.8 Inter‐ and intracellular crosstalk analysis using cellphone DB and scMLnet

Cellphone DB was employed to explore the cell–cell interactions between malignant cells and niche cell subtypes based on the ligand–receptor pairs.^16^ The receptors and ligands with a mean expression level > 1 and a *p* value < 0.01 were considered positive ligand–receptor pairs. GGplot2, psych, qgraph, igraph and tidyverse R packages were used to visualize the intratumor crosstalk network.

scMLnet was used to explore the intercellular and intracellular signalling network between CAFs, T cells and malignant cells. The analysis details of scMLnet were elucidated as previously.^17^, ^18^ LogFC > 2 and *p*_valj < 0.05 were considered as the cutoff criteria.

### 2.9 Differential expression and enrichment analyses

We calculated the differentially expressed genes (DEGs) in cell subgroups using the findmarker function provided by Seurat. Avg_logFC > 0.5 and *p*_val_adj < 0.05 were considered as the cutoff criteria. The ClusterProfiler R package was used to perform GO and KEGG analyses. GSEA was performed to show the enriched gene set based on the expression of each gene. We used GSVA R packages to accomplish the GSVA analysis.

### 2.10 Trajectory analysis

We used the Monocle 2.0 package (v 2.10.0) to analyse single‐cell trajectories and determine the continuous process of T cell exhaustion. We used the top 1000 differentially expressed genes in CD8+ Tcyto cells and Tex cells to sort cells in pseudotime order. Branch expression analysis modelling (BEAM analysis) was used to analyse branch fate‐related genes based on pseudotime analysis.

### 2.11 Classification of molecular subgroups by consistent clustering

The ConsensusClusterPlus package in R software was applied for consistent clustering to determine subgroups of EC samples from TCGA. The Euclidean squared distance metric and the *K*‐means clustering algorithm were used to classify samples into *k* clusters with *k* = 2 to *k* = 8. Eighty percent of the samples were selected in each iteration, and the results were compiled after 50 iterations. We determined the optimal number of clusters by constructing a consistent cumulative distribution function (CDF) graph and the delta region graph.

### 2.12 Construction of a prognostic predictive signature

Univariate Cox regression analysis was conducted to identify the prognostic value of the DEGs in vCAFs, and genes with a *p* value < 0.01 were considered statistically significant. The regression coefficient (*β*) was determined by performing a multivariate Cox regression analysis, and the risk score = (βmRNA1 * expression level of mRNA1) + (βmRNA2 * expression level of mRNA2) + … + (βmRNAn * expression level of mRNAn). Patients with survival data were divided into high‐ and low‐risk groups based on the median risk score.

### 2.13 Independence of the prognostic gene signature from other clinical characteristics

Univariate and multivariate Cox proportional hazard regression analyses were performed to determine whether the predictive ability of the prognostic model was independent of conventional clinical characteristics. A bilateral *p* value < 0.05 was considered statistically significant. The hazard ratios (HRs) and 95% confidence intervals were calculated.

### 2.14 HE and IHC analysis

After deparaffinization, slides were hydrated in alcohol and endogenous peroxidase activity was quenched for 20 min in 3% hydrogen peroxide. Antigen epitope retrieval was induced by high temperature and pressure. To examine the expression pattern of candidate antibodies in EC tissues, sections were immunostained with primary antibodies overnight at 4°C, The secondary antibody used for immunostaining was biotin‐conjugated anti‐rabbit immunoglobulin (Wuhan antgene biotechnology Co.,Ltd, Cat. No. ANT058).

The following antibodies were used to detect specific proteins: anti‐EPCAM (rabbit, 1:4000, Abcam, Cat. No. ab282457), anti‐α‐SMA (rabbit, 1:2000, Abcam, Cat. No. Ab5694), anti‐PDPN (rabbit, 1:4000, Abcam, Cat. No. Ab236529), anti‐IGF1 (rabbit, 1:100, ABclone, Cat. No. A0830), anti‐MYH11 (rabbit, 1:1000, Abcam, Cat. No. Ab133567) and anti‐CD74 (rabbit, 1:200, Abcam, Cat. No. ab108393).

### 2.15 Statistical analysis

Continuous variables are summarized as the means ± standard deviations (SD). Differences between groups were compared using the Wilcox test with R software. The significance of differences in survival time was calculated using the log‐rank test with a threshold of a *p* value < 0.05. Kaplan–Meier curves were plotted to show the differences in survival times.

## 3 RESULTS

### 3.1 Single‐cell transcriptomic profiling of human endometrial cancer

**FIGURE 1:**
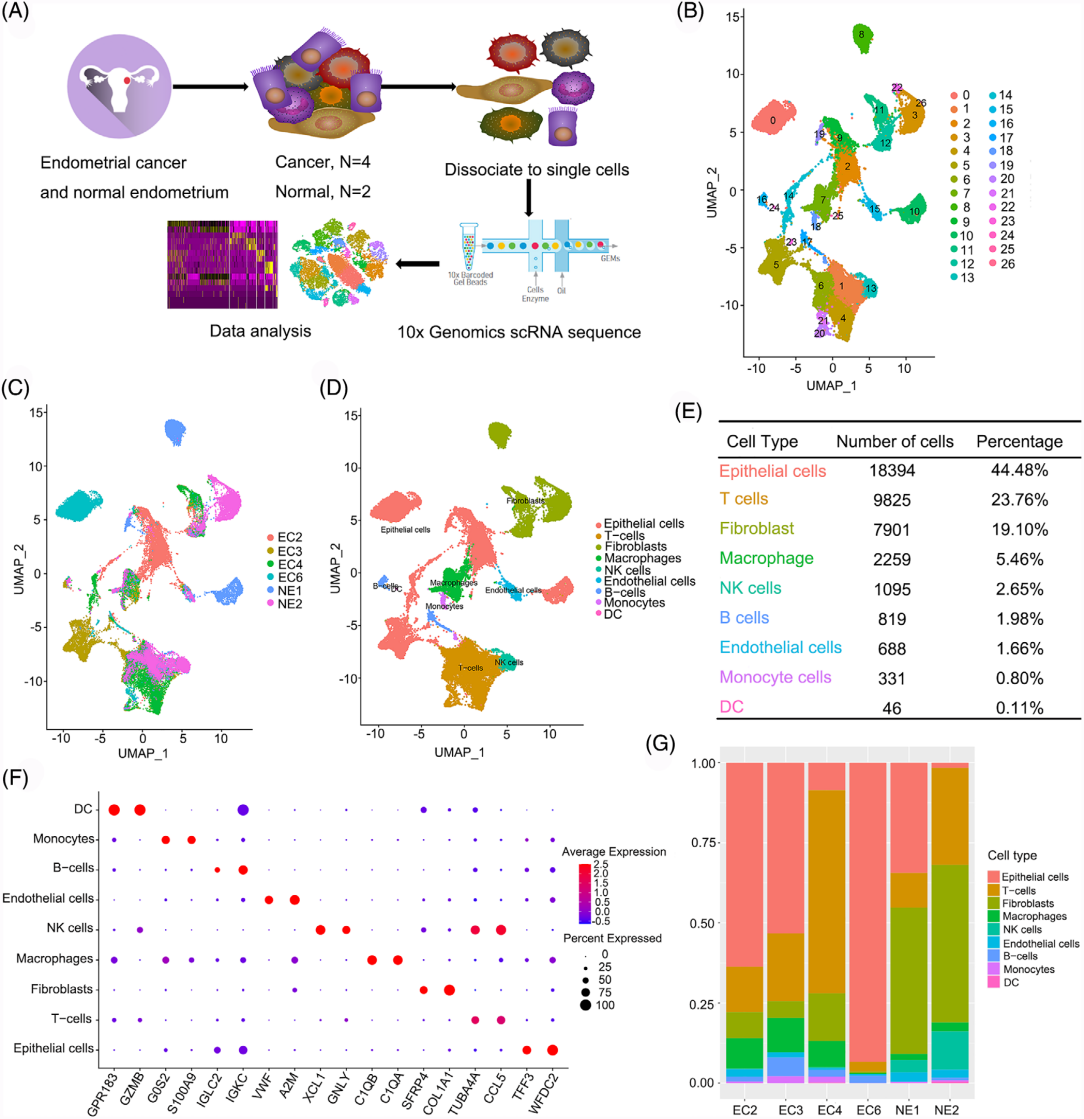
Comprehensive overview of human endometrial cancer. (A) Schematic diagram of scRNA‐seq analysis workflow; (B) UMAP plotting of the 41,358 cells showing 27 cell clusters; (C) The sample origin of the cells; (D) The distinct cell types identified by marker genes; (E) The number of cells in each cell type; (F) Bubble plots showing marker genes for 9 distinct cell types; (G) Bar plots showing the proportion of cell types in each sample

Four endometrial tumours and two normal endometrial tissues were collected to explore the complex cellular diversity and relevant molecular characteristics (Figure 1A). The specific clinical pathological features and H&E staining of tissues from the enrolled patients are shown in Table S1 and Figure S1. Two experienced pathologists checked and approved the sections to determine the consensus diagnosis. After filtering with strict standards, 41,358 single cells with a median of 1313 genes per cell were retained for further bioinformatics analysis. The numbers of cells captured in each sample are shown in Table S2. We conducted principal component analysis (PCA) and uniform manifold approximation and projection (UMAP) following gene expression normalization to reduce the dimensionality. Then, graph‐based clustering was used to divide the cells into 26 clusters (Figure 1B) that were annotated for nine cell types with specific expression of marker genes: epithelial cells (18,394 cells, 44.5%, marked with KRT8 and KRT18), T cells (9825 cells, 23.8%, marked with CD2, CD3D and CD3E), fibroblasts (7901 cells, 19.1%, marked with COL1A1, SFRP4, ACTA2, COL1A2), macrophages (2259 cells, 5.5%, marked with CD163, CD86, C1QB and C1QA), natural killer (NK) cells (1095 cells, 2.6%, marked with GNLY, NCAM1, and XCL1), endothelial cells (688 cells, 1.7%, marked with A2M, VWF and ENG), B cells (819 cells, 2.0%, marked with IGKC and CD79A), monocytes (331 cells, 0.8%, marked with S100A8 and S100A9) and dendritic cells (DC) (46 cells, 0.1%, marked with GPR183 and PLD4) (Figures 1D–F, S2G, Table S3). Conspicuously, the proportion of each cell type varied substantially among different samples, indicating the existence of intertumoral heterogeneity in endometrial cancer (Figure 1C,G).

### 3.2 Identification of malignant epithelial cells in endometrial cancer

**FIGURE 2:**
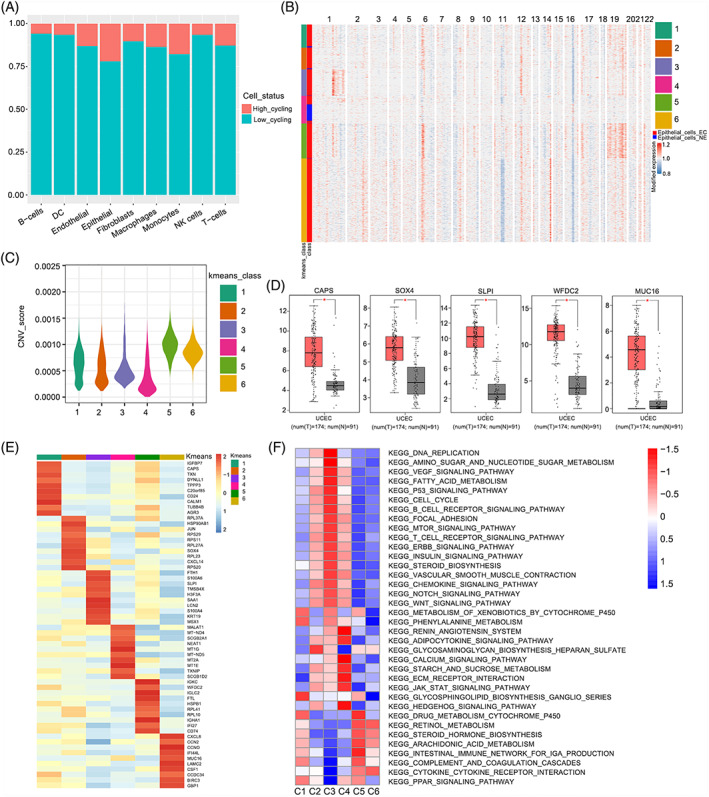
Transcriptomic heterogeneity of malignant cells in EC. (A) The cell cycling status of distinct cell types; (B) The heatmap of the relative expression density of genes on each chromosome by comparing the tumour cell genome with a series of normal cell reference genomes; (C) The CNV scores of each *k*‐means class; (D) The expression level of marker gene in TCGA dataset, (*p < 0.05); (E) Heatmap of DEGs in each *k*‐means class; (F) Differences in pathway activity (scored per cell by GSVA) in 6 epithelial cell sub‐clusters

We first explored the cycling status of all cell subtypes to identify malignant epithelial cells and found that epithelial cells included a large proportion of high cycling cells, indicating that the epithelial cells were actively undergoing mitosis (Figure 2A). An analysis of copy number variation (CNV) was employed to distinguish malignant epithelial cells from epithelial cells isolated from normal samples (Figure S3). Then, we re‐clustered the epithelial cells into six classes and calculated the CNV scores for each class (Figure 2B,C). We referred to C4 as a nonmalignant epithelial class for two reasons: (1) C4 had the lowest CNV score, and (2) C4 cells were primarily derived from normal samples. Therefore, C1, C2, C3, C5 and C6 were considered malignant epithelial classes. The differential expressed genes (DEGs) revealed that each class had unique transcriptomic characteristics (Figure 2E, Table S4). We also explored the representative marker genes of each class, such as CAPS (C1), SOX4 (C2), SLPI (C3), WFDC2 (C5) and MUC16 (C6) in TCGA and GTEx databases (Figure 2D). The expression levels of these oncogenes were higher in tumour samples, consistent with the results of our single‐cell sequencing analysis. Gene set variation analysis (GSVA) showed the distinct functions of six classes, such as the PPAR signalling pathway (C1), glycosaminoglycan biosynthesis heparan sulfate (C2), DNA replication and P53 signalling pathway (C3), calcium signalling pathway (C4), intestinal immune network for IgA production (C5) and cytokine‐cytokine receptor interaction (C6) (Figure 2F). Overall, the malignant epithelial cells of endometrial cancer may play diverse roles in tumour progression.

### 3.3 Malignant cells might induce an immunosuppressive microenvironment in endometrial cancer

**FIGURE 3:**
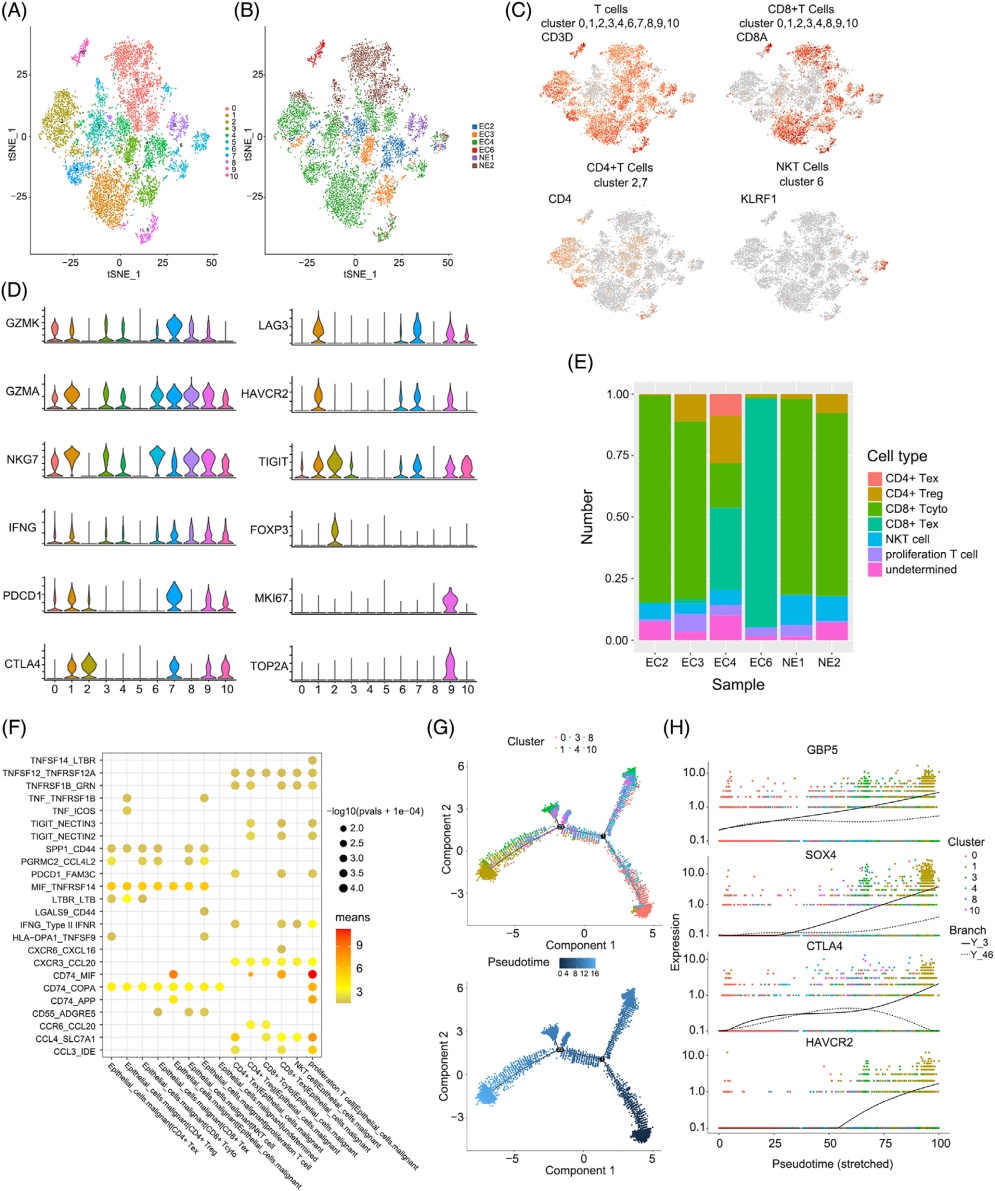
Profiling of immune microenvironment in EC and intratumoral crosstalk with malignant cells. (A) t‐SNE plotting of the T cells showing 11 cell clusters; (B) The sample origin of the cells; (C) t‐SNE plots of marker genes for each cell type as indicated; (D) Violin plots of selected cytotoxicity, proliferation, and suppressive genes in distinct T cell subclusters; (E) Bar plots showing the proportion of cell types in each sample; (F) Interaction analysis showing enriched receptor‐ligand pairs in subsets of T cells and malignant cells; (G) Trajectory of differentiation from CD8+ Tcyto into Tex predicted by monocle 2; (H) Significantly up‐regulated genes in the differentiation process coloured by cell clusters

The immune microenvironment of endometrial cancer is considered crucial to the prognosis, and overall survival varies substantially in patients with different immune subtypes.^19^ We classified T cells into 11 clusters to explore the inherent heterogeneity of the immune microenvironment, and the t‐SNE plot shows the distribution among cancer and normal samples (Figure 3A,B). All clusters had a relative expression of CD3D except Cluster 5 (Figure 3C), and the DEG analysis showed that Cluster 5 did not contain a marker gene (Figure S4A); therefore, we identified it as an undetermined cluster (Figure S4B). Clusters 0, 1, 3, 4, 8, 9 and 10 were associated with the marker CD8A, Clusters 2 and 7 were associated with the marker CD4, and Cluster 6 was considered NKT cells labelled with KLRF1 (Figure 3C). Then, Clusters 0, 3, 4 and 8 were named cytotoxic CD8^+^ T cells (CD8^+^Tcyto) marked with GZMK, GZMA, NKG7 and IFNG. Clusters 1, 7 and 10 represent activated T cells with exhausted features based on the expression of effector (IFNG and NKG7), cytotoxic (GZMK and GZMA) and immune checkpoint molecules (PDCD1, CTLA4, and LAG3); thus, we designated them experienced T cells (Tex).^20^ Cluster 2 was characterized by a high level of FOXP3, indicating that it was a regulatory T cell (CD4^+^ Treg) population. Cluster 9 was referred to as proliferation T cells, which was marked with MKI67 and TOP2A (Figure 3D). We concluded that the immune microenvironment varies substantially between different people, suggesting that the individual heterogeneity has to be considered concerning immune‐based therapy (Figure 3E). Proliferating T cells also showed exhausted characteristics based on the expression of PDCD1, CTLA4, LAG3, HAVCR2 and TIGIT (Figure 3D). We performed inter‐cellular interaction analyses based on ligand‐receptor pairs to explore the frequent communication between different subtypes of T cells and malignant cells. We conclude that proliferation T cells maintain the most frequent interactions with other subtypes of T cells (Figure S4C). In addition, malignant cells may induce an immunosuppressive microenvironment due to greater interactions with T cells presenting exhausted characteristics through ligand‐receptor pairs such as TNF‐ICOS, indicating that blocking TNF‐ICOS binding may affect the interaction of CD4^+^ Tregs with malignant cells and might be an effective therapeutic target for endometrial cancer (Figure 3F). To further explore the intracellular gene regulatory networks, an integrated multilayer network between malignant epithelial cells and T cells was constructed through scMLnet (Table S5). Proliferation T cells maintained close communications with malignant epithelial cells which was consistent with our Cellphone DB results. Furthermore, malignant epithelial cells may accelerate the proliferation of CD4^+^Tregs and CD8^+^Tex through CALM1/FAS binding, and then activate the downstream nuclear factor kB (NF‐kB) transcript factors, such as NFKBIA, NFKB2 and REL, regulating downstream CCND2, CDK6 and TRAF1 expression (Figure S5). These results suggested that malignant epithelial cells played an important role in the formation of immunosuppressive microenvironment in EC.

A trajectory analysis was applied using CD8^+^ Tcyto cells and Tex cells to investigate the dynamic expression pattern under the exhaustion of T cells (Figure S4D). The pseudotime results showed that CD8^+^ Tcyto cells ultimately transited to Tex cells after experiencing three diverging cell fates (Figure 3G). Along the trajectory, the levels of exhaustion markers such as CTLA‐4 and HAVCR2 were gradually increased during the transition. In addition, GBP5 and SOX4 displayed a similar trend to the exhaustion markers, indicating that they might represent potential markers of T cell depletion (Figure 3H).

### 3.4 Cancer‐associated fibroblasts show distinct characteristics

**FIGURE 4:**
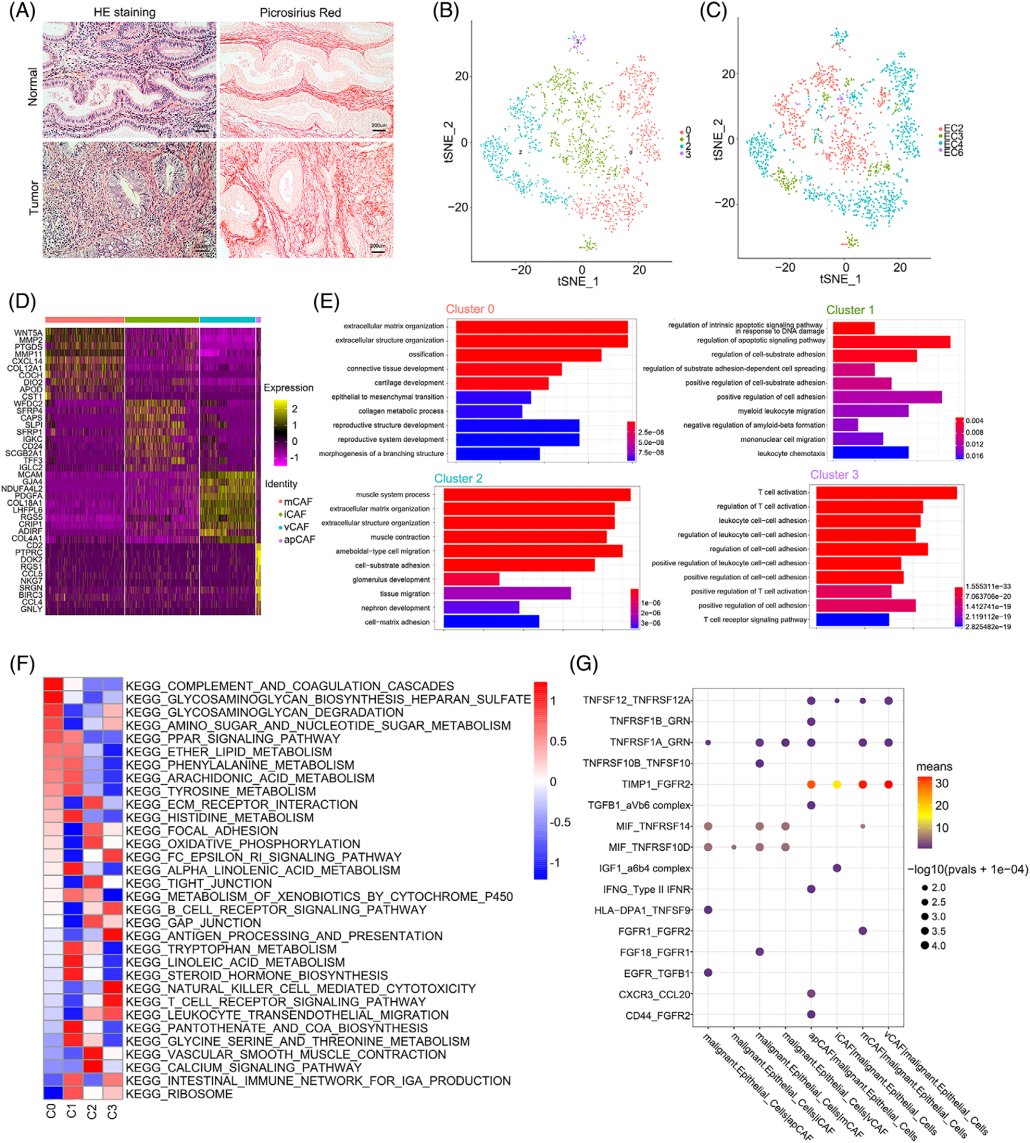
Distinct cancer‐associated fibroblasts subpopulations detected in human EC. (A) H&E, picrosirius red staining in EC and normal tissues; (B) t‐SNE plotting of the cancer‐associated fibroblasts (CAFs) showing 4 cell clusters; (C) The sample origin of the cells; (D) Heatmap showing the top 10 DEGs (Wilcoxon test) for each cluster; (E) GO analysis of DEGs in distinct CAF subclusters; (F) GSVA analysis revealing the hallmark pathways in distinct CAF subclusters; (G) Dot plot showing receptor‐ligand pair analysis of the interactions between malignant cells and distinct cell types

An intense desmoplastic reaction has been observed in many malignant tumours, such as pancreatic ductal adenocarcinoma (PDAC), cervical cancer and colorectal cancer.^21^, ^22^, ^23^ Picrosirius red and IHC staining for α‐SMA revealed that the desmoplastic reaction was also prominent in endometrial cancer (Figure 4A). We generated 2059 cancer‐associated fibroblasts (CAFs) from four endometrial cancer samples, which were further clustered into four subclusters (Figure 4B,C). All clusters were positive for the expression of classic fibroblast markers such as ATCA2, COL1A1, COL3A1, and THY1 (Figure S6A). The heterogeneity of CAFs was obviously detected in the t‐SNE dimensionality reduction plot. In addition, each cluster showed an exclusive expression pattern, suggesting that it may perform a unique function in the tumour ecosystem (Figure 4D, Table S6). We compared the genes between CAFs and fibroblasts in normal endometrium (NE) to initially explore the functions, and Gene Ontology (GO) and Kyoto Encyclopedia of Genes and Genomes (KEGG) enrichment analyses revealed that the up‐regulated genes in CAFs were enriched for extracellular matrix organization, response to wounding, angiogenesis, antigen processing and presentation, indicating the distinct characteristics of CAFs (Figure S6D).

Cells in Cluster 0, accounting for 37.2% of the CAFs, featured an extracellular matrix (ECM) signature, such as glycoprotein (PDPN), structural protein (COL12A1), matricellular proteins (FBLN2 and SOX6) and matrix modifying enzymes (LOXL1 and MMP2) (Figures 4D and S6B). In addition, the GO enrichment analysis showed that the upregulated genes were enriched in the terms extracellular matrix organization, extracellular structure organization and collagen metabolic process (Figure 4E). Thus, we defined Cluster 0 as matrix CAFs (mCAF‐C0‐PDPN). Cluster 1, which comprised 34.7% of CAFs, expressed SLPI, IGF1, CD24, CXCL12 and TFF3 at high levels (Figures 4D and S6B). The GO terms enriched in this cluster were related to myeloid leukocyte migration, mononuclear cell migration and leukocyte chemotaxis (Figure 4E). Therefore, we designated Cluster 1 as an inflammatory CAF population (iCAF‐C1‐IGF1). Cluster 2 was characterized by high levels of MYH11, GJA4, RGS5, ESAM, MCAM and EPAS1 (Figures 4D and S6B). The GO enrichment analysis also showed that muscle system process, muscle contraction and tissue migration were enriched in this cluster (Figure 4E). Meanwhile, we explored the markers (CD34, CDH5, PECAM1 and TIE1) of endothelial cells to eliminate the disturbance of endothelial cells and proved the low expression levels (Figure S6C). Therefore, we accordingly named Cluster 2 vascular CAFs (vCAF‐C2‐MYH11). As the cluster with the fewest cells, Cluster 3 was characterized by antigen‐presentation signatures, such as major histocompatibility complex II (MHC‐II) genes (CD74, HLA‐DPA1, HLA‐DPB1 and HLA‐DQB1) (Figures 4D and S6B). Moreover, enriched GO terms were mainly involved in immunomodulation, such as regulation of T cell activation and regulation of leukocyte cell–cell adhesion (Figure 4E). Thus, we designated Cluster 3 as antigen‐presenting CAFs (apCAF‐C3‐CD74). Interestingly, cells in Cluster 3, which were mainly derived from EC4, expressed immune checkpoint molecules (PDCD1, CTLA‐4, LAG3, HAVCR2, TIGIT and ICOS) at high levels, indicating that they may contribute to an immunosuppressive microenvironment in EC4 (Figure S6E). Gene set variation analysis (GSVA) also showed that each cluster had specific biological functions that were consistent with the enrichment results (Figure 4F). In addition, we further verified the presence of those clusters in EC samples using IHC staining (Figure S7).

### 3.5 Ligand–receptor analysis and multilayer signalling network indicates frequent communication between malignant cells and CAFs

Malignant cells maintained frequent communication with other cells in the tumour microenvironment to accomplish their biological functions. A ligand–receptor analysis was conducted as described above to explore the internal crosstalk between malignant and stromal cells. We concluded that various degrees of interactions existed between malignant cells and different types of CAFs. Interestingly, the interactions between malignant and stromal cells were more frequent than those between malignant cells (Figure S6F). We sought to identify effective cytokines, such as interleukins, growth factors and chemokines, mediating the functions of malignant and stromal cells. Notably, the highest expression of TIMP1 and FGFR2 was observed in malignant cells and different types of CAFs (Figure 4G). In addition, the IGF1/α6β4 complex pair was enriched in the interactions between iCAFs and malignant cells, consistent with the finding that malignant subclusters displayed activation signatures such as DNA replication and the mTOR signalling pathway (Figure 2F). Multilayer signalling network revealed that CAFs showed more ligands than malignant epithelial cells (Table S7). The transcription factors such as SMAD7, KLF4 and TBX2 and downstream proto‐oncogenes such as MYC, MET and CDK1 were regulated by the ligand–receptor binding of CAFs and malignant epithelial cells (Figure S8). These results indicated that the frequent intratumor crosstalk between different cell types contributes to the malignant progression of EC.

### 3.6 vCAFs facilitated the malignant progression of endometrial cancer and was a poor prognostic factor

**FIGURE 5:**
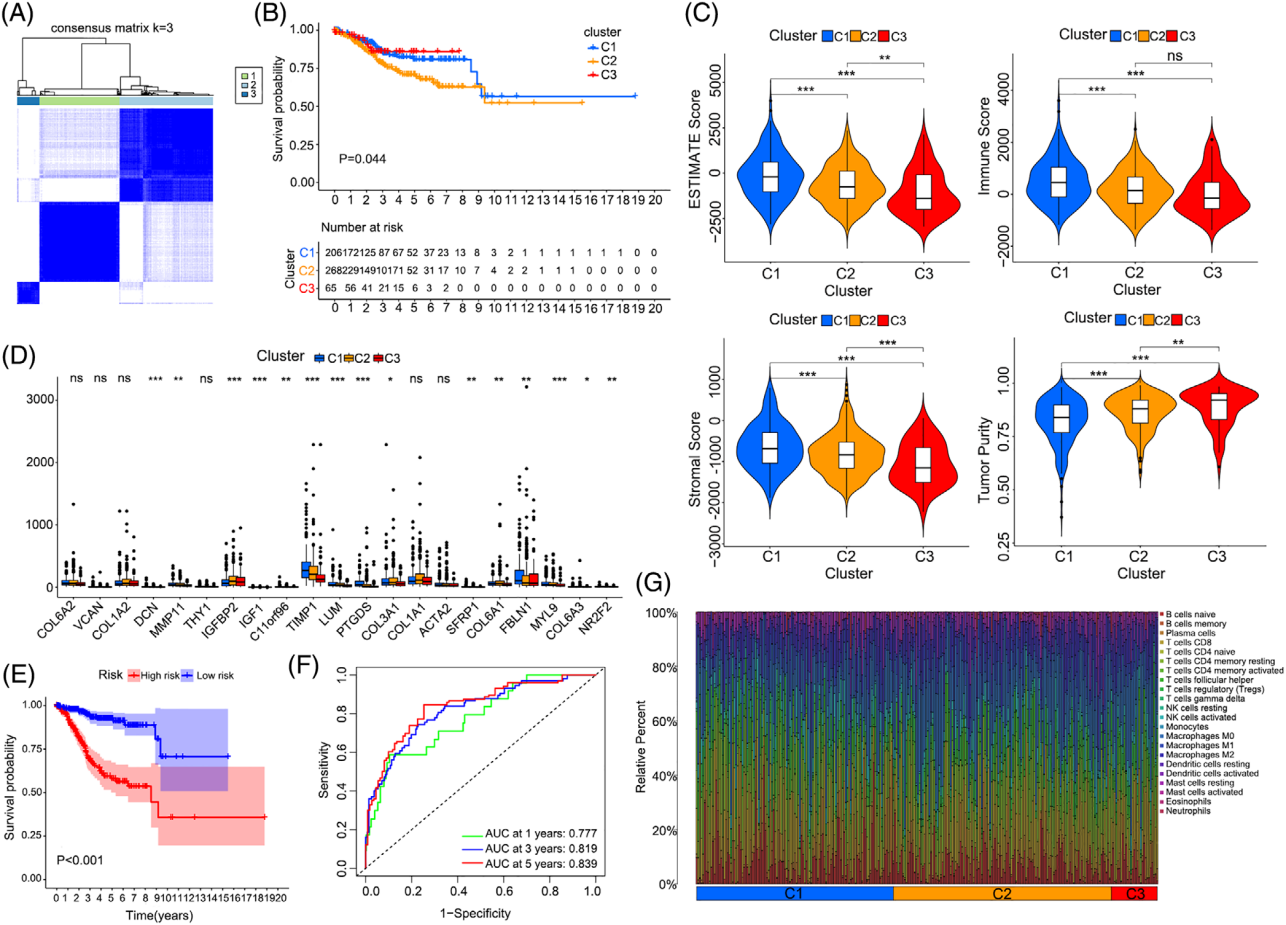
Prognostic significance of vCAF. (A) Heatmap showing the clustering result for the value of consensus clustering based on the vCAF markers; (B) Kaplan‐Meier survival analysis of tumour samples grouped in A; (C) Violin plots showing the estimated scores of TME in each cluster, (*p < 0.05, **p < 0.01, ***p < 0.001); (D) The expression level of classic stroma markers in each cluster; (E) Kaplan‐Meier survival curve of the prognostic model for TCGA EC patients; (F) Time‐dependent ROC curves of the prognostic model for 1‐,3‐ and 5‐year overall survival in EC; (G) The infiltrating immune cells in different cluster

We next analysed public endometrial carcinoma (EC) data from TCGA (https://cancergenome.nih.gov/) to investigate the clinical value of gene expression patterns in different types of CAFs. Due to the heterogeneity of CAFs, the DEGs of four CAFs were applied for consistent clustering, respectively (Figures 5A and S9A), and the survival analysis showed that the three clusters determined by vCAF resulted in significant differences in survival (Figure 5B). The ESTIMATE score was derived by combined Stromal score and Immune score to predict the tumour purity.^24^ The C3 cluster with the lowest stroma score calculated using the Estimate R package was associated with prolonged survival (Figure 5C). In addition, classic stromal markers, such as COL6A3, COL6A1, COL3A1, C11orf96, TIMP1, LUM and PTGDS, were expressed at relatively lower levels in the C3 cluster than in the other clusters (Figure 5D). However, the clinicopathological information indicated that the C3 cluster had worse differentiation and later stages (Figure S9B). Univariate and subsequent multivariate Cox regression analyses were applied to construct the prognostic model and better explore the prognostic value of vCAFs (Figure S9C). Based on the results of Kaplan–Meier survival analysis, patients in the low‐risk group experienced a significantly longer OS than those in the high‐risk group (Figure 5E), and we also performed a ROC curve analysis to evaluate the predictive accuracy of our prognostic model (Figure 5F). Moreover, the prognostic model was indicated to be an independent factor for conventional clinical characteristics (Figure S9D). Interestingly, we found that the tumour microenvironment of the C3 cluster contained more infiltrating CD8^+^ T cells, consistent with a previous study showing that the stromal component may impede immune cell infiltration (Figures 5G and S9E).^25^, ^26^ These results revealed that the existence of vCAFs was a poor prognostic factor for EC patients.

## 4 DISCUSSION

Tumour heterogeneity is one of the reasons that leads to treatment failure.^27^ In this study, scRNA‐seq was applied to comprehensively delineate the heterogeneity and intratumor crosstalk of human ECs at single‐cell resolution. This granularity of analysis facilitated identification of subtype of CAF (vCAF) as an independent characteristic of a worse prognosis. We also determined that the characteristics of the immune microenvironment varied between different patients with EC. In addition, malignant cells may induce an immunosuppressive microenvironment by interacting with exhausted T cells, potentially driving resistance to PD‐1/PD‐L1‐based immunotherapy.^28^

The TME, also termed the tumour stroma or tumour mesenchyme, is composed of fibroblasts, inflammatory cells, blood vessels, extracellular matrix (ECM) and basement membrane. These components interact with tumour cells to ensure the relative homeostasis of the TME. Previous studies have elucidated that the desmoplastic reaction in the TME is an unfavourable prognostic indicator for patients with colorectal cancer, intrahepatic cholangiocarcinoma and endometrial cancer.^29^, ^30^, ^31^ However, the heterogeneity of CAFs makes this conclusion controversial because different types of CAFs may have opposite functions.^32^, ^33^ Hutton et al. distinguished two pancreatic fibroblast lineages with distinct functions using mass cytometry, concluding that CD105^pos^ fibroblasts are tumour permissive, whereas CD105^neg^ fibroblasts suppress tumour growth in a manner dependent on adaptive immunity.^34^ Most previous studies have focused on the protumorigenic functions of CAFs based on coculture or coimplantation with cancer cells in vitro and in vivo, while this topic remains unelucidated in EC.^35^, ^36^, ^37^ The roles of different types of CAFs in disease progression and prognosis must be clarified, and future studies will address this critical point.

The advent of single‐cell sequencing has facilitated a deeper understanding of the complex TME at single‐cell resolution. Four types of CAFs with different characteristics were defined in our study through single‐cell sequencing. By combining our results with those from public databases, we identified that vCAFs were an independent risk factor for EC in part because they restrained the infiltration of immune cells. Min Zhang et al. verified the existence of CD146^+^ vCAFs that exhibited close interactions with intrahepatic cholangiocarcinoma (ICC) cells through the IL‐6/IL‐6R interaction.^11^ Although we highlighted the role of vCAFs in the progression of EC, important roles for other types of CAFs could not be excluded. Recent studies have reported that mCAFs, iCAFs and apCAFs play crucial roles in tumour aggression and overall survival through various mechanisms.^38^, ^39^, ^40^ However, the results need to be validated in an independent dataset in future.

Taken together, our findings revealed a comprehensive transcriptomic landscape of human EC and confirmed the prognostic significance of vCAFs, which may provide deeper insights into cancer therapy.

## AUTHOR CONTRIBUTIONS

Zhicheng Yu and Jun Zhang: conception of the work, data analysis and interpretation, drafting the article, critical revision of the article and final approval of the version to be published. They contributed equal to this study. Qi Zhang, Sitian Wei and Rui Shi: data collection, critical revision of the article and final approval of the version to be published. Lanfen An and Rong Zhao: data collection, data analysis and interpretation. Richard Grose: data analysis, critical revision of the article and final approval of the version to be published. Hongbo Wang and Dilu Feng: conception of the work, critical revision of the article and final approval of the version to be published. All authors contributed to the article and approved the submitted version.

## CONFLICT OF INTEREST

All other authors have no conflicts to disclose.

## Supporting information


**FIGURE S1** H&E staining, EPCAM and α‐SMA IHC staining of EC samples and normal tissues for the scRNA‐seq analysis

**FIGURE S2** Cellular quality and cellular identity of all 41,358 cells. (A) Boxplots showing the median read counts (transcript, nUMI) and expressed genes (nGene) of all samples; (B) Scree plot show top30 PCs of principle component analysis which were used in downstream analysis; (C) Boxplots showing the threshold value to remove the multi‐cellular barcode; (D) Removal of the mitochondrial genes; (E) The volcano plot showing the 2000 highly variable genes; (E) Violin plots showing marker genes for 9 distinct cell types

**FIGURE S3** Copy number variations (CNVs) evaluated per cell by InferCNV. Two normal‐derived epithelial clusters were used as control group

**FIGURE S4** Intratumoral crosstalk and exhaustion process of T cells. (A) Heatmap showing the top 8 marker genes of 11 T cell clusters; (B) UMAP plotting of the 7 T cell clusters; (C) The intratumoral crosstalk of subsets of T cells with malignant cells; (D) Heatmap showing dynamic expression under the exhaustion of CD8+ T cells

**FIGURE S5** Multilayer networks between malignant epithelial cells and T cells produced by scMLnet. The multilayer signalling network consists of four layers: ligand layer, receptor layer, TF layer and target gene layer

**FIGURE S6** Characteristics and intratumoral crosstalk of distinct CAFs with malignant cells. (A) UMAP plots showing the classic markers of fibroblasts; (B) Violin plots showing the marker genes of distinct subsets of CAFs; (C) Violin plots showing the marker genes of endothelial cells; (D) The GO and KEGG results of DEGs between CAFs and normal fibroblasts; (E) Violin plots showing the expression of immune check points in cluster 3; (F) The heatmap and network plot showing the intra‐tumoral crosstalk of distinct CAFs with malignant cells.

**FIGURE S7** Validation of distinct CAF subclusters by IHC staining

**FIGURE S8** Multilayer networks between malignant epithelial cells and CAFs produced by scMLnet. The multilayer signalling network consists of four layers: ligand layer, receptor layer, TF layer and target gene layer

**FIGURE S9** Consensus clustering of EC molecular subgroups based on vCAF DEGs. (A) Cumulative distribution function (CDF) curve; (B) The clinical difference between C1 and C3 clusters; (C) Forrest plot of the univariate analysis association with overall survival; (D) Forrest plot of the univariate and multivariate association of the prognostic model and clinicopathological characteristics with overall survival; (E) Bar plots showing the infiltrating immune cells in different cluster

**TABLE S1** Clinical characteristics of EC patients and control samples profiled by scRNA‐seq and validated by staining in this study, related to Figure 1

**TABLE S2** Numbers of cells captured in each sample

**TABLE S3** Top 30 differential expression genes for the 9 clusters

**TABLE S4** Top 30 differential expression genes for the 6 epithelial subclusters

TABLE S5

**TABLE S6** Top 30 differential expression genes for the 4 CAF subclusters

TABLE S7

## Data Availability

Data sharing is not applicable to this article as no new data were created or analyzed in this study.
