# Supplementary figures and images for: Single‐cell sequencing reveals the heterogeneity and intratumoral crosstalk in human endometrial cancer
Source: Cell Prolif. 2022 May 13;55(6):e13249. doi: 10.1111/cpr.13249 (PMC9201371; doi:10.1111/cpr.13249)

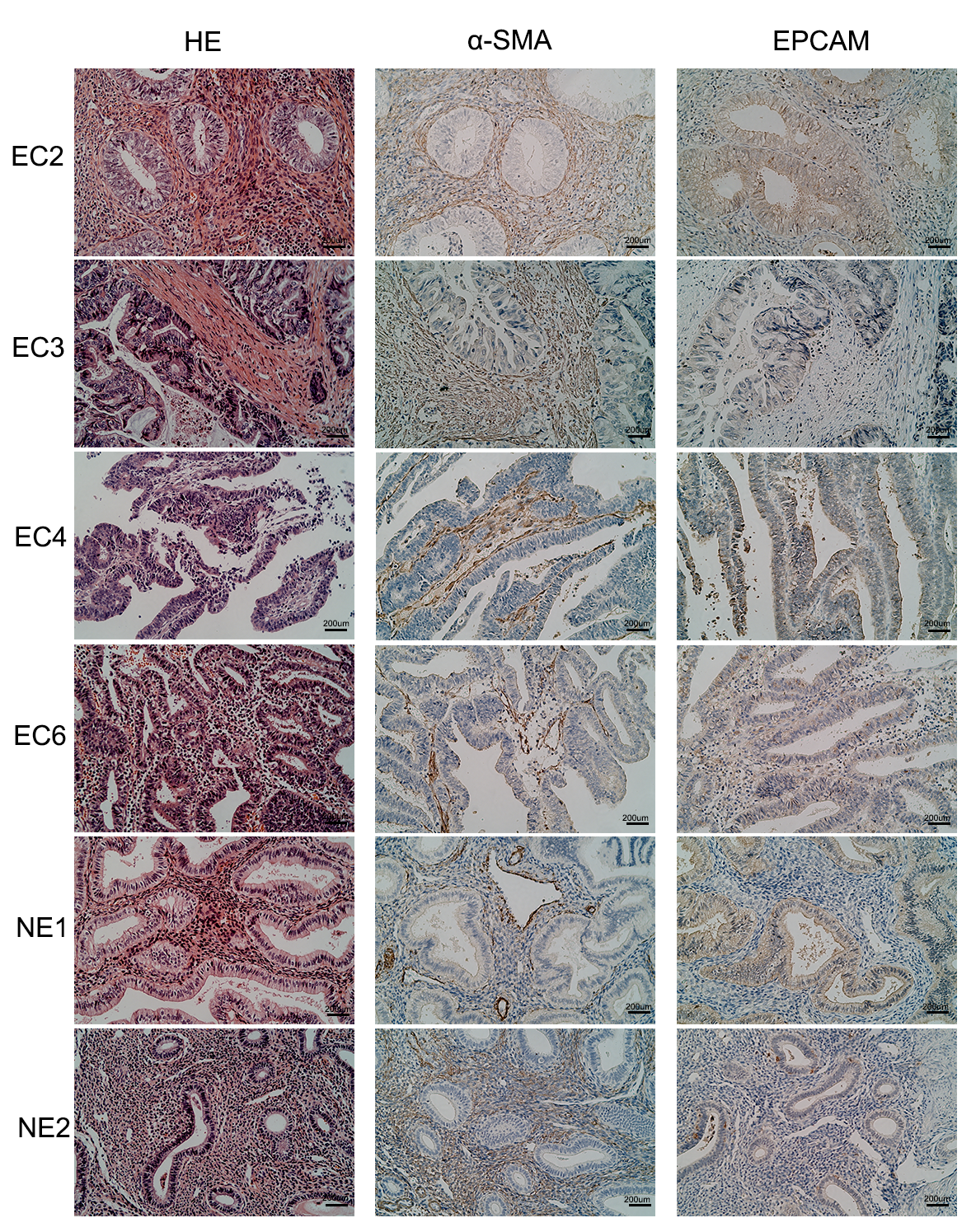

Supplement: Supplementary file 1 — FIGURE S1 H&E staining, EPCAM and α‐SMA IHC staining of EC samples and normal tissues for the scRNA‐seq analysis [file CPR-55-e13249-s014.tif]

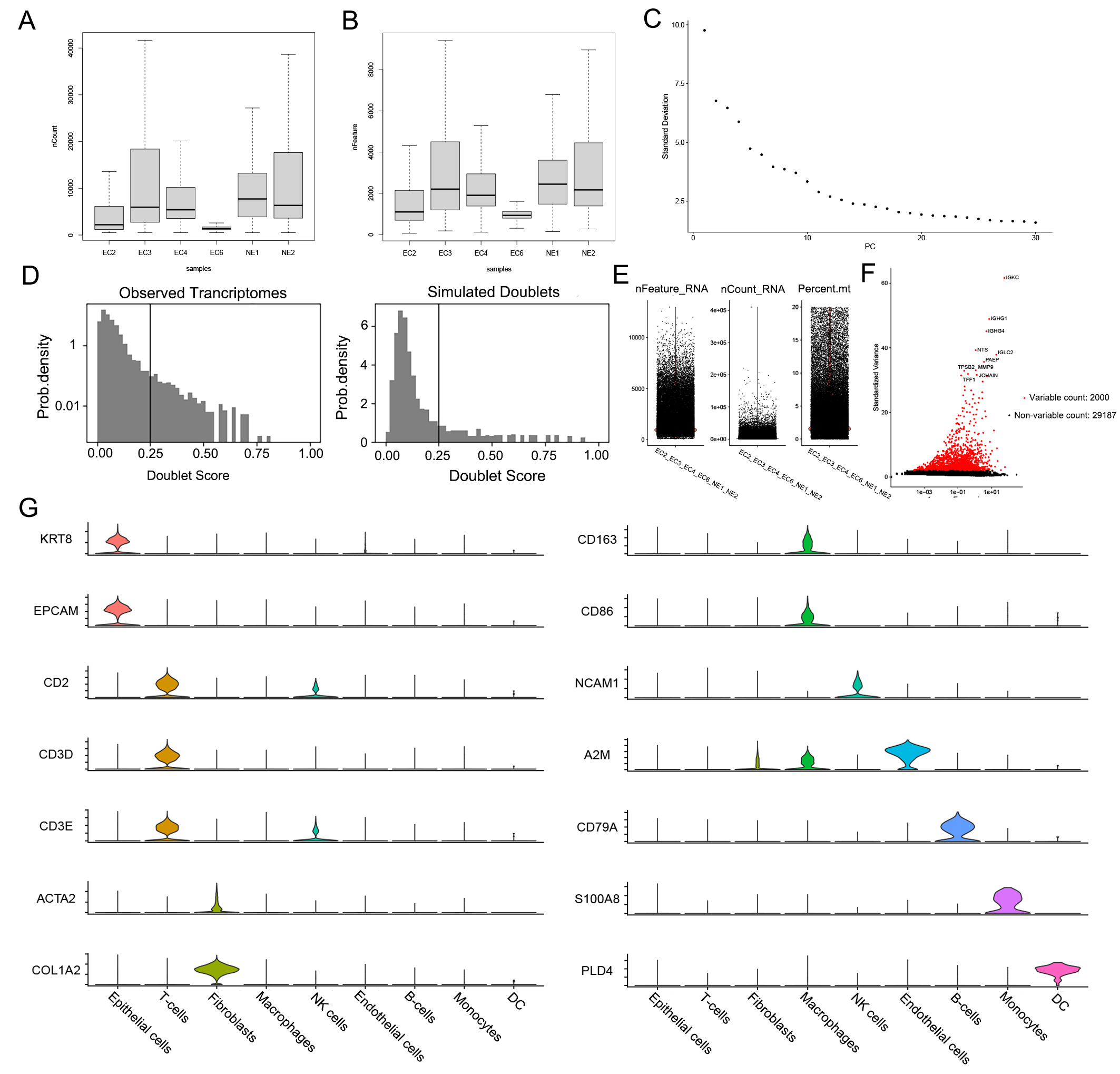

Supplement: Supplementary file 2 — FIGURE S2 Cellular quality and cellular identity of all 41,358 cells. (A) Boxplots showing the median read counts (transcript, nUMI) and expressed genes (nGene) of all samples; (B) Scree plot show top30 PCs of principle component analysis which were used in downstream analysis; (C) Boxplots showing the threshold value to remove the multi‐cellular barcode; (D) Removal of the mitochondrial genes; (E) The volcano plot showing the 2000 highly variable genes; (E) Violin plots showing marker genes for 9 distinct cell types [file CPR-55-e13249-s009.tif]

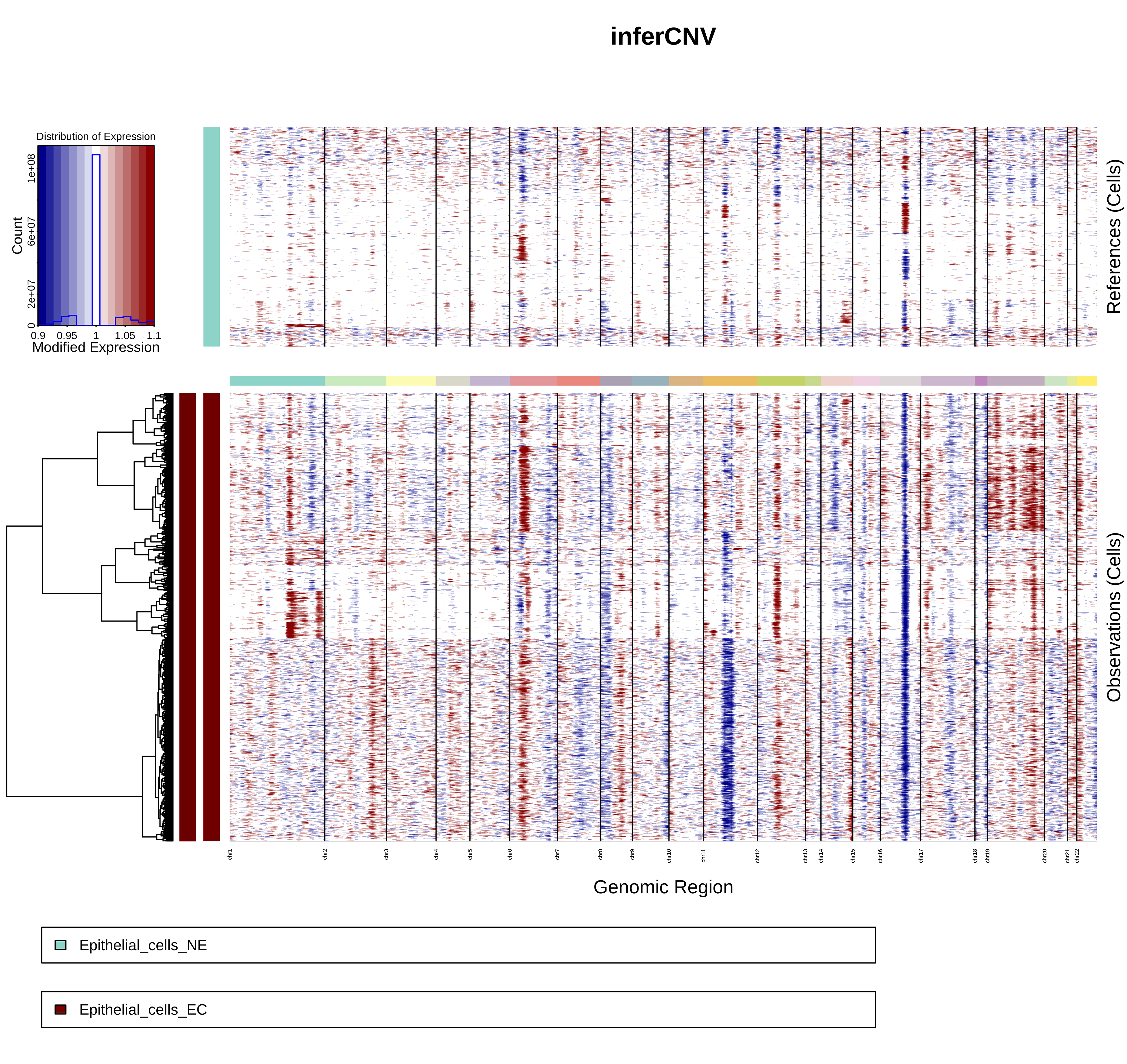

Supplement: Supplementary file 3 — FIGURE S3 Copy number variations (CNVs) evaluated per cell by InferCNV. Two normal‐derived epithelial clusters were used as control group [file CPR-55-e13249-s004.tif]

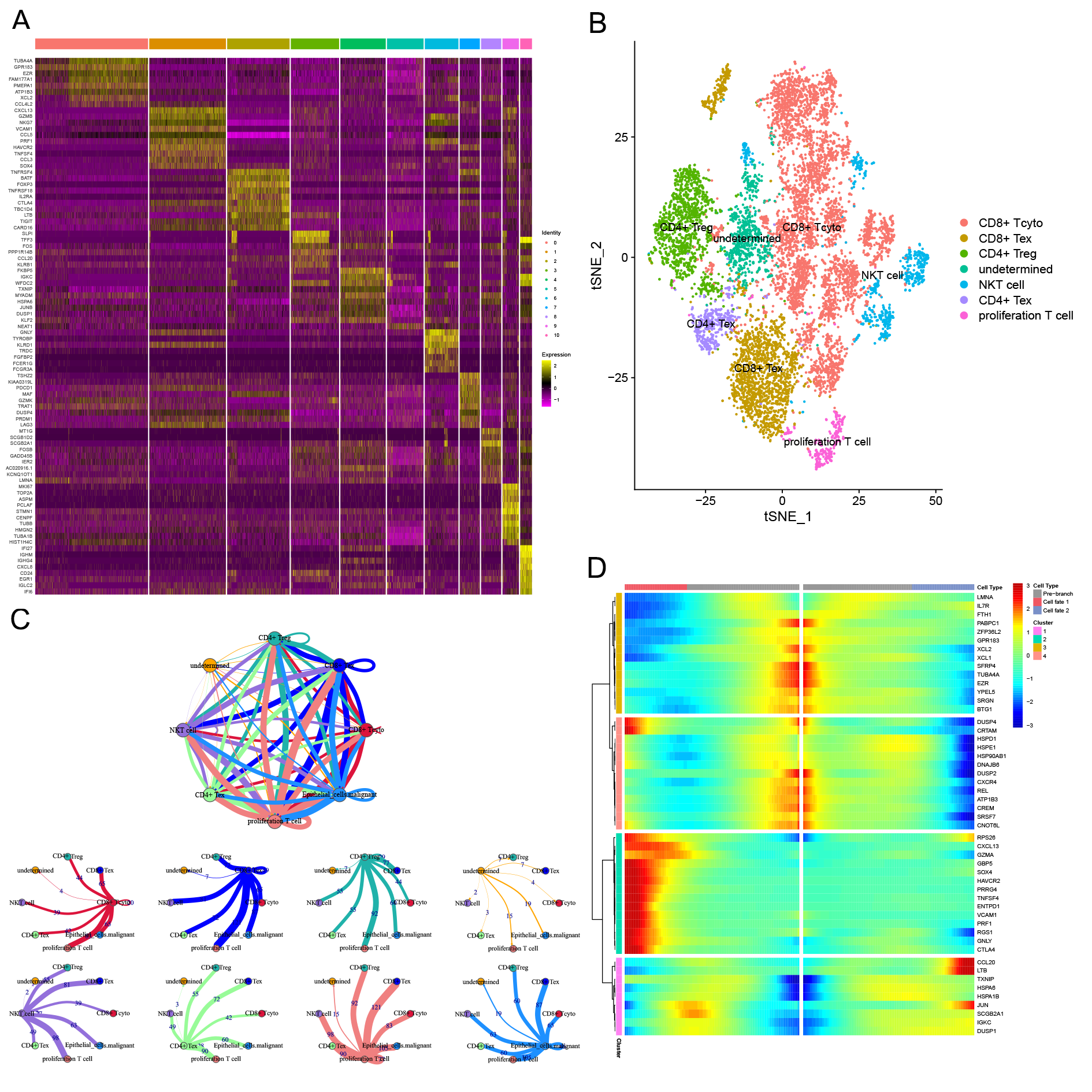

Supplement: Supplementary file 4 — FIGURE S4 Intratumoral crosstalk and exhaustion process of T cells. (A) Heatmap showing the top 8 marker genes of 11 T cell clusters; (B) UMAP plotting of the 7 T cell clusters; (C) The intratumoral crosstalk of subsets of T cells with malignant cells; (D) Heatmap showing dynamic expression under the exhaustion of CD8+ T cells [file CPR-55-e13249-s007.tif]

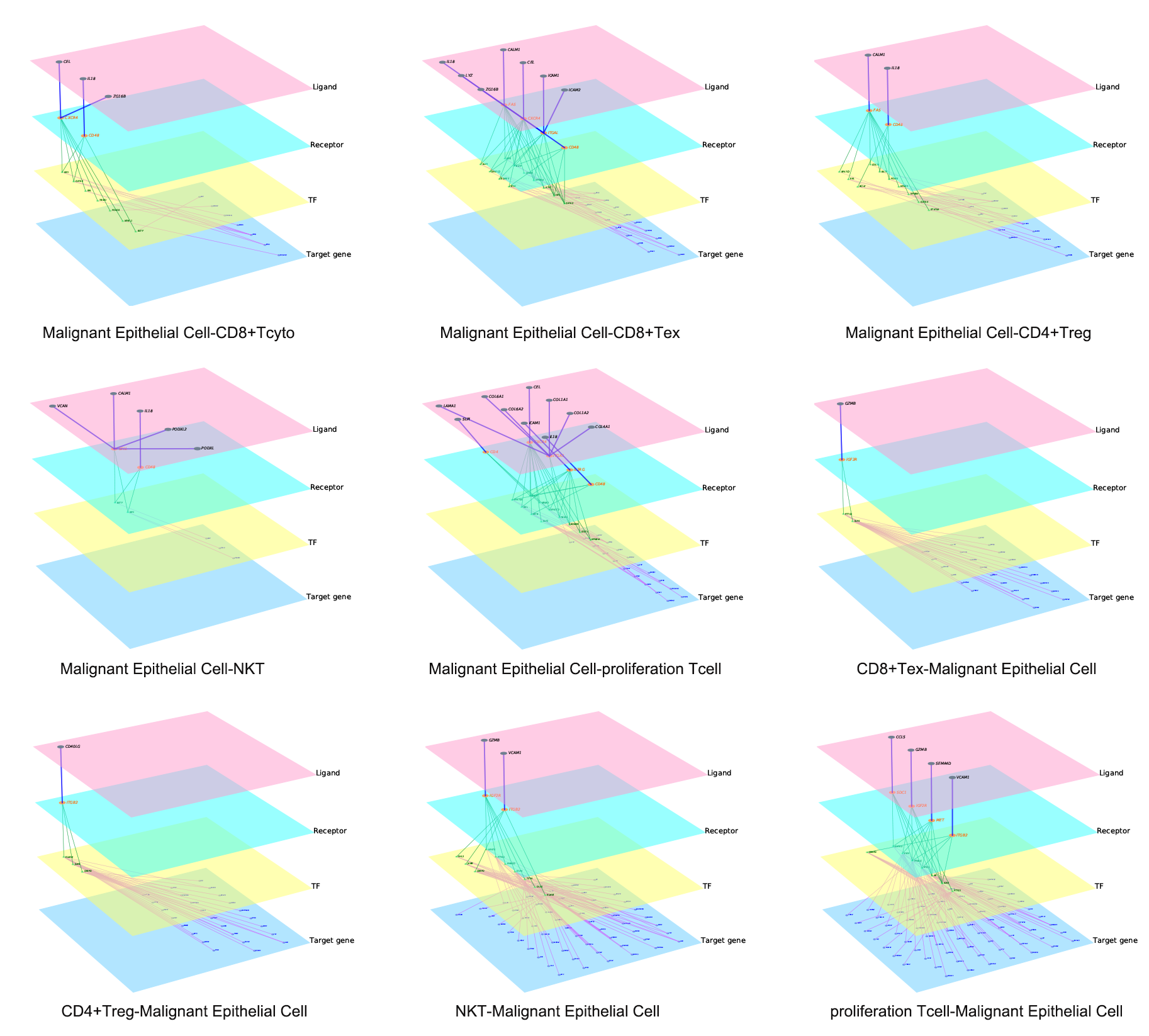

Supplement: Supplementary file 5 — FIGURE S5 Multilayer networks between malignant epithelial cells and T cells produced by scMLnet. The multilayer signalling network consists of four layers: ligand layer, receptor layer, TF layer and target gene layer [file CPR-55-e13249-s001.tif]

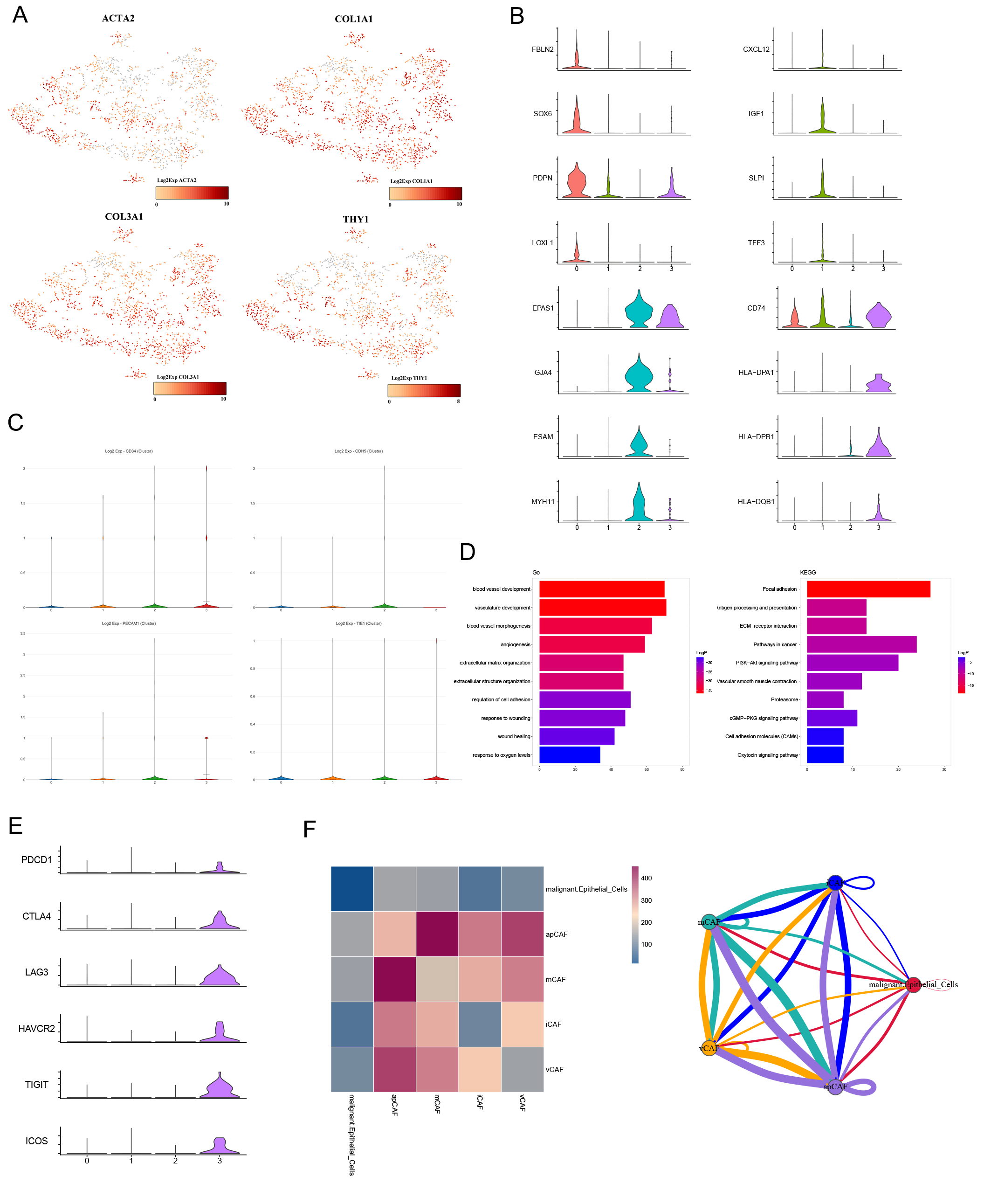

Supplement: Supplementary file 6 — FIGURE S6 Characteristics and intratumoral crosstalk of distinct CAFs with malignant cells. (A) UMAP plots showing the classic markers of fibroblasts; (B) Violin plots showing the marker genes of distinct subsets of CAFs; (C) Violin plots showing the marker genes of endothelial cells; (D) The GO and KEGG results of DEGs between CAFs and normal fibroblasts; (E) Violin plots showing the expression of immune check points in cluster 3; (F) The heatmap and network plot showing the intra‐tumoral crosstalk of distinct CAFs with malignant cells. [file CPR-55-e13249-s015.tif]

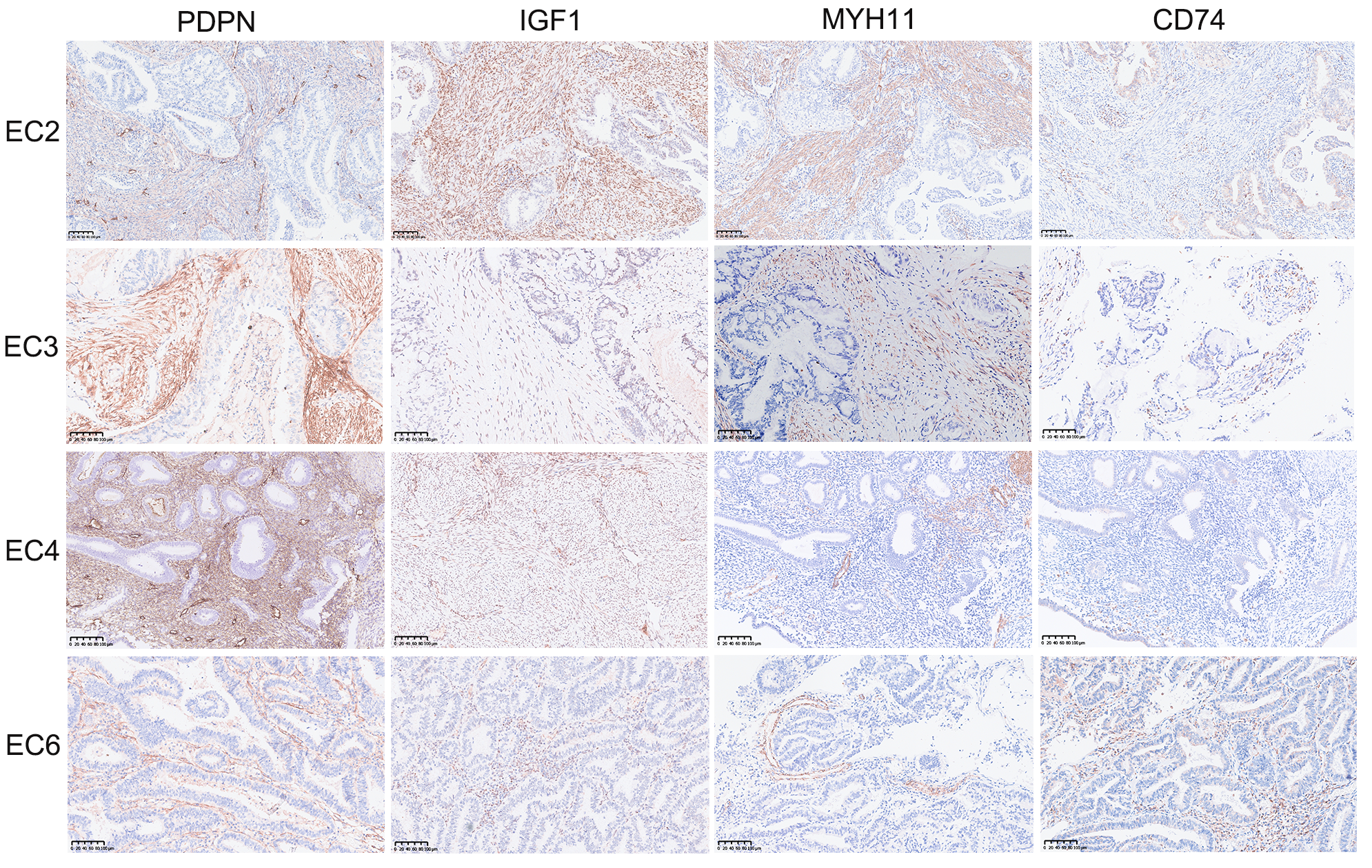

Supplement: Supplementary file 7 — FIGURE S7 Validation of distinct CAF subclusters by IHC staining [file CPR-55-e13249-s006.tif]

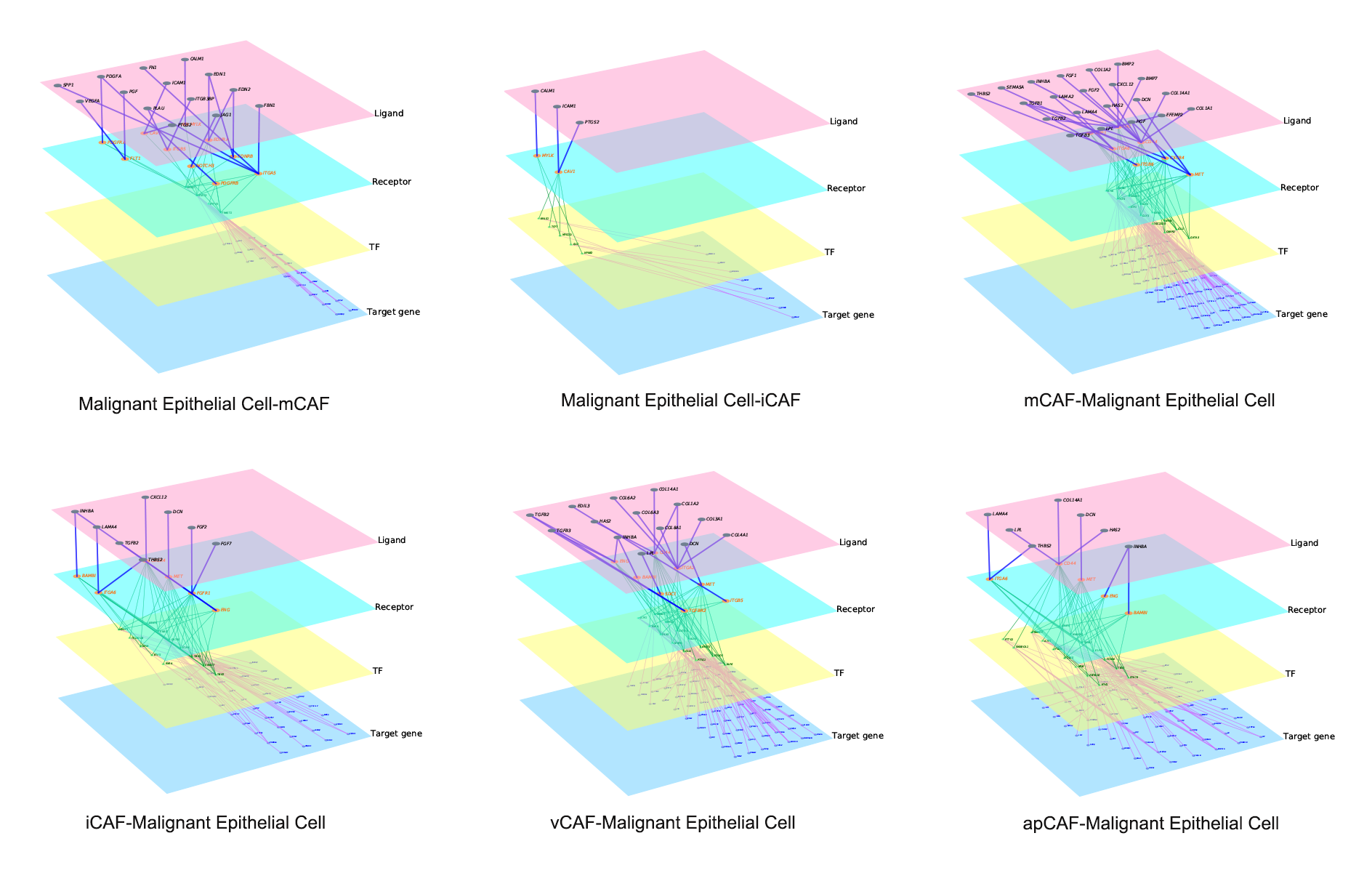

Supplement: Supplementary file 8 — FIGURE S8 Multilayer networks between malignant epithelial cells and CAFs produced by scMLnet. The multilayer signalling network consists of four layers: ligand layer, receptor layer, TF layer and target gene layer [file CPR-55-e13249-s002.tif]

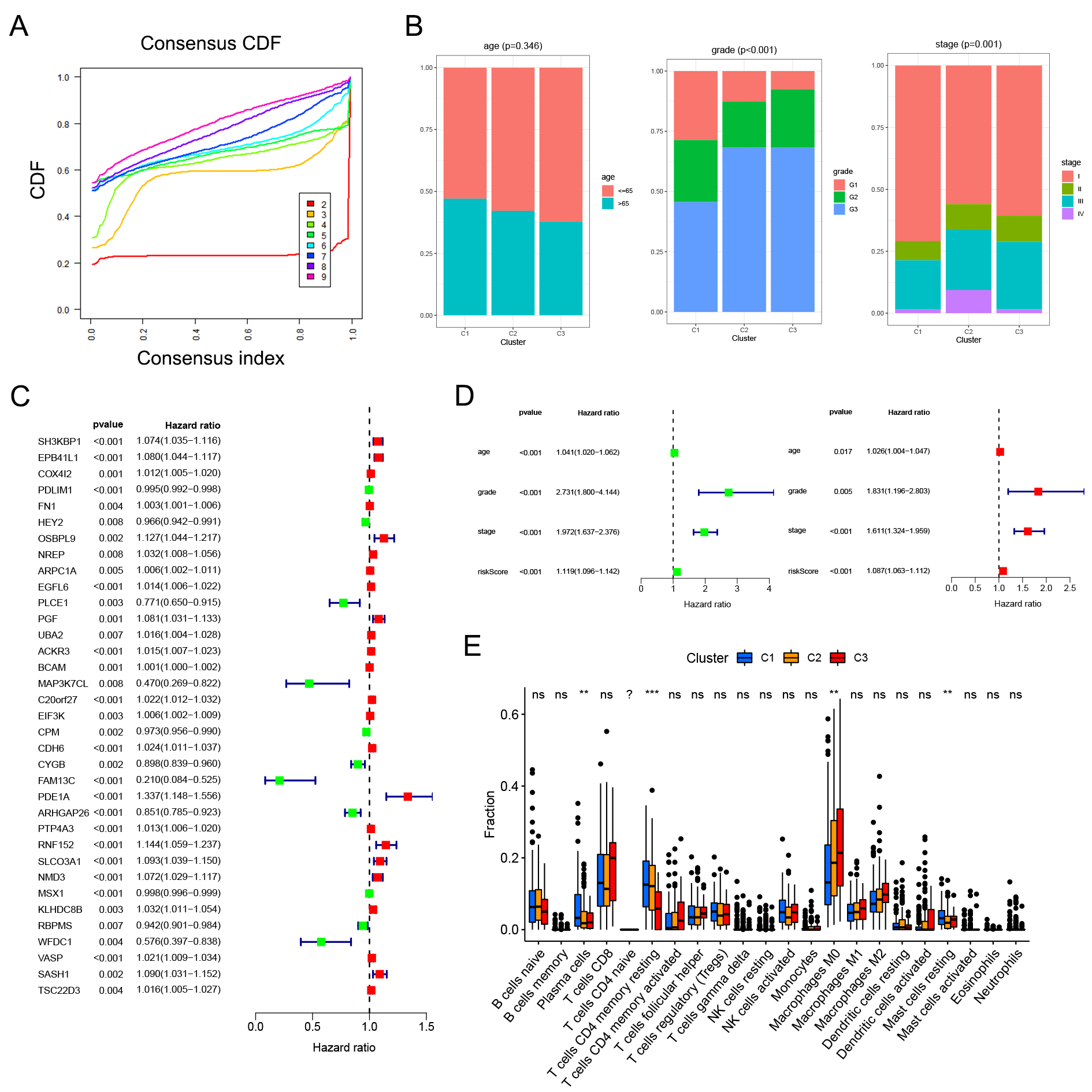

Supplement: Supplementary file 9 — FIGURE S9 Consensus clustering of EC molecular subgroups based on vCAF DEGs. (A) Cumulative distribution function (CDF) curve; (B) The clinical difference between C1 and C3 clusters; (C) Forrest plot of the univariate analysis association with overall survival; (D) Forrest plot of the univariate and multivariate association of the prognostic model and clinicopathological characteristics with overall survival; (E) Bar plots showing the infiltrating immune cells in different cluster [file CPR-55-e13249-s013.tif]
